# Online Influence and Sentiment of Fitness Tweets: Analysis of Two Million Fitness Tweets

**DOI:** 10.2196/publichealth.8507

**Published:** 2017-10-31

**Authors:** Theodore Vickey, John G Breslin

**Affiliations:** ^1^ College of Engineering & Informatics National University of Ireland Galway Galway Ireland; ^2^ Department of Kinesiology Point Loma University San Diego, CA United States

**Keywords:** Twitter, physical activity, mobile fitness apps, fitness tweet classification, sentiment

## Abstract

**Background:**

Publicly available fitness tweets may provide useful and in-depth insights into the real-time sentiment of a person’s physical activity and provide motivation to others through online influence.

**Objective:**

The goal of this experimental approach using the fitness Twitter dataset is two-fold: (1) to determine if there is a correlation between the type of activity tweet (either workout or workout+, which contains the same information as a workout tweet but has additional user-generated information), gender, and one’s online influence as measured by Klout Score and (2) to examine the sentiment of the activity-coded fitness tweets by looking at real-time shared thoughts via Twitter regarding their experiences with physical activity and the associated mobile fitness app.

**Methods:**

The fitness tweet dataset includes demographic and activity data points, including minutes of activity, Klout Score, classification of each fitness tweet, the first name of each fitness tweet user, and the tweet itself. Gender for each fitness tweet user was determined by a first name comparison with the US Social Security Administration database of first names and gender.

**Results:**

Over 184 days, 2,856,534 tweets were collected in 23 different languages. However, for the purposes of this study, only the English-language tweets were analyzed from the activity tweets, resulting in a total of 583,252 tweets. After assigning gender to Twitter usernames based on the Social Security Administration database of first names, analysis of minutes of activity by both gender and Klout influence was determined. The mean Klout Score for those who shared their workout data from within four mobile apps was 20.50 (13.78 SD), less than the general Klout Score mean of 40, as was the Klout Score at the 95th percentile (40 vs 63). As Klout Score increased, there was a decrease in the number of overall workout+ tweets. With regards to sentiment, fitness-related tweets identified as workout+ reflected a positive sentiment toward physical activity by a ratio of 4 to 1.

**Conclusions:**

The results of this research suggest that the users of mobile fitness apps who share their workouts via Twitter have a lower Klout Score than the general Twitter user and that users who chose to share additional insights into their workouts are more positive in sentiment than negative. We present a novel perspective into the physical activity messaging from within mobile fitness apps that are then shared over Twitter. By moving beyond the numbers and evaluating both the Twitter user and the emotions tied to physical activity, future research could analyze additional relationships between the user’s online influence, the enjoyment of the physical activity, and with additional analysis a long-term retention strategy for the use of a fitness app.

## Introduction

Physical activity can reduce the risk for many different types of chronic diseases and can help people maintain a healthy weight. Although this knowledge is widely known, adults and children in many countries do not get recommended amounts of physical activity [1]. Recent advances in physical activity monitoring now provide researchers with unparalleled opportunities to increase and improve our understanding of the health benefits of physical activity by assessing daily quantities of activity, patterns, and trends [2], as well as the real-time sentiment of physical activity. Research suggests that technology is one factor that has contributed to the increase in sedentary behavior and decrease in physical activity, but it has also led to a number of innovative physical activity interventions [1].

One such innovation is through the use of mobile fitness apps and the sharing of one’s workout through a social network. This paper will focus on the collection of self-reported fitness data through a mobile fitness app that is then shared with one’s social network via Twitter. The dataset of these tweets along with other connected datasets of demographic information allows for a number of analyses, including but not limited to the potential influence of such tweets and the sentiment of these tweets. By combining the digital traces as people interact through mobile phones and emerging technology may now provide novel methods to assess a range of factors objectively and with minimal expense and burden to participants [3]. This paper will review both the potential online influence and the sentiment of the shared fitness tweets.

Social media has changed the way society is exposed to information [4]. Social networking sites such as Twitter have developed into increasingly useful platforms for the general public to share thoughts, ideas, and opinions. Twitter is a free social networking platform that is widely used around the world by businesses and individuals and is considered one of the most widely used microblogging platforms with 328 million monthly active users with more than 1 billion unique monthly visits to sites with embedded tweets with a mission to “to give everyone the power to create and share ideas and information instantly, without barriers” [5]. Twitter users can rapidly and directly share with and respond to a massive audience, using messages of 140 characters or less. With the creation and introduction of newly developing technologies such as Twitter, new opportunities to obtain global health data that may circumvent the limitations of traditional data sources used in population health and physical activity research are now available [3].

At the same time, these publicly shared data are resulting in vast and growing user-contributed repositories of data [6]. Twitter provides user-generated data that can be collected and analyzed to examine opinions around health-related foci, including discussions about physical activity, alcohol and marijuana use, depression, and suicide [3]. From a health-promotion standpoint, these data can be useful to measure participants’ dependence on social support, given that exercisers today are just as, if not more, likely to seek motivation and validation from social media—in particular, Twitter—than their in-person friends and family members [7]. Because it is possible to glean precise information from tweets, including the time of the tweet and location of the user, this suggests that the 140-character messages could be predictive in other areas, such as the types of physical activity that users engage in and where and when they engage in these activities.

Using Twitter integration with mobile fitness apps can be a helpful tool for obtaining descriptive and predictive real-time shared health information in a noninvasive way. New and innovative cloud-based data collection and analysis tools may aid research efforts because they can yield a large collection of tweets in a short period of time. They may also be useful for longitudinal data collection [8]. The link between publicly available health and fitness data sources is made possible as more users publicly share their self-collected data from devices and apps through social media services such as Twitter [9]. An enhanced understanding of mobile fitness apps and the sharing of physical activity through one’s social network, the different types of measurement properties, and the subsequent generated data are critical to furthering our understanding of daily physical activity.

Sentiment analysis is a classification process, the primary focus of which is to predict the polarity of words and to then classify these words as positive, negative, or neutral with the aim of identifying attitude and opinions [10]. Specific to Twitter, sentiment analysis is the task of automatically identifying and extracting subjective information from tweets. This method of data analysis has received increasing attention from the Web-mining community [11]. Although Twitter provides extremely valuable insight into publicly shared opinions, it also provides new big data challenges, including the processing of massive volumes of data and the identification of human expressiveness within short text messages [11]. Much of the existing research on textual information processing has been focused on the mining and retrieval of factual information, with little research on the processing of opinions [12].

The mining of Twitter for data provides a rich database of information on people’s thoughts and sentiments about a myriad of health topics, including physical activity. Analysis of social networks data using Twitter has become a powerful tool that is currently being used to answer research questions across the health spectrum, including local and national flu surveillance [13], the sharing of information between cancer patients [14], marijuana usage among teens [15], and drug safety surveillance [16]. This paper represents, to the best of our knowledge, the first analysis of shared tweets from mobile fitness apps specific to physical activity. A significant proportion of tweets contained nonneutral sentiments regarding the shared physical activity of the four mobile apps featured in this research.

The ability to evaluate the sentiment of an individual immediately after a bout of physical activity has been completed can be powerful. A typical tweet might include the type of exercise performed, the duration and intensity of that exercise, and how the person felt during and after the activity. If the sentiment is negative (eg, “Just hiked to the top of Mt Pisgah. Took me 2 hours and I’m completely exhausted. Don’t think I’ll do that again! #myfitnesspal”), a coach or trainer can intervene and modify the activity accordingly. Finding exercise that is enjoyable and of the appropriate intensity is an important precursor to long-term adherence. Behavioral researchers suggest that one’s emotions can profoundly affect individual behavior and decision making [17]. Simply stated, a tweet can be a window into real emotion provided in real time.

Other research reported that when fitness promoters initiated a #PlankADay challenge on Twitter—which was designed to encourage core-strengthening exercise—72% of users participated for at least 30 days straight and at the end of the challenge reported an increased enjoyment of the activity and expressed interest in continuing to do abdominal exercise [18]. This indicates that Twitter and other social networks can be useful in spreading exercise awareness and encouraging positive exercise behaviors. Together, this information can facilitate research on how technology can be used to monitor and motivate physical activity and how online social networks may play a role in physical activity promotion and adherence. Identifying the types of people who use mobile fitness apps and finding ways to track what they do and motivate them to continue to engage in physical activity is a form of data mining for this “customer base.”

## Methods

### Collection of Tweets

After a review of online tools that could collect and manage tweets, an open-source program called TwapperKeeper was deemed appropriate as the Twitter data-collection tool. TwapperKeeper is a Web app designed to collect social media data via Twitter for long-term archival and analysis. The app uses a Twitter-enabled application program interface (API) that acts as an interface between the Twitter search function and a cloud database for tweet storage [19].

For this research, we chose four mobile fitness apps based on their availability on iPhone, the ability of the mobile fitness app to share workout information through Twitter, and the fact that they targeted beginner versus experienced exercisers. The research team used these criteria to narrow possible choices and reviewed additional academic research for previously used apps, researched publicly available reviews on different mobile fitness apps, interviewed both developers and users of mobile fitness apps to obtain their input, and met as a group to finalize the selected mobile fitness apps to study [20].

The four apps chosen were Endomondo, Nike+, RunKeeper, and DailyMile. Tweets were then collected from the mobile fitness apps using the following hashtags: #endomondo, #nikeplus, #runkeeper, and #dailymile. These were used because these apps automatically attach these hashtags to a tweet to indicate it has come from that particular mobile fitness app. It is through these hashtags that common themes or information can be grouped within Twitter.

Data collection using TwapperKeeper continued for 184 days. During this period, 2,856,534 user-generated mobile fitness app tweets were collected in 23 different languages. The Twitter data in this study was public, and the research was deemed exempt from human subjects review. This research was approved by the institutional review board of the National University of Ireland Galway in Galway, Ireland.

Two analyses were completed on a dataset of collected tweets from four mobile fitness apps. The first was to measure the online influence of Twitter users through their Klout Score. The second was to measure the sentiment of physical activity-related tweets.

### Analysis 1: Measuring Online Influence

One important factor to consider when analyzing tweets to report physical activity is the credibility and authority of the person sending the tweets. Previous data collectors have looked at a Twitter user’s number of followers, although researchers discovered that monitoring retweets and the messages themselves are a better predictive tool [21].

Websites such as Klout have developed the means to determine a user’s reach or influence on social media. The Klout Score is the measurement of a person’s overall online influence, with scores ranging from 1 to 100; higher scores represent a wider and stronger sphere of influence. Scores greater than 50 are rare [22]. A Klout Score places less emphasis on a user’s number of followers and number of tweets, but rather measures the extent to which the user’s content is retweeted [23]. One’s influence on Twitter can be difficult to measure accurately. Klout uses more than 3600 features that capture the online social network activity of the user to conduct the influence analysis and calculate the Klout Score [24]. The Klout Score allows for tailored statistical analysis of social media usage and is tangible proof of the effect of the Internet on a person’s lifestyle [25]. With regards to influence, Internet users perceived a mock Twitter page with a high Klout Score as more credible than the same page with a moderate or low Klout Score [26].

Online influence services such as Klout are in the process of scoring millions, eventually billions, of people on their level of influence. To proponents, the measurement of online influence is an inspiring tool that encourages the democratization of influence, where one no longer must be a celebrity, politician, or media personality to be considered influential.

#### Recruitment

For this experimental approach, the user’s Klout Score—a measure of their online influence—was used to compare shared physical activity levels from mobile fitness apps.

In this experiment, we examined the sharing of fitness tweets from within mobile fitness apps (Nike+, RunKeeper, DailyMile, and Endomondo) and analyzed the data based on the participant’s gender and online influence, as measured by their Klout Score. We identified two types of activity tweets from dataset: workout tweets, which included what was generated by the mobile fitness app, and workout+ tweets, which included the same information as a workout tweet but also contained user-created communication. We hypothesized that those with a higher Klout Score would share fewer minutes of activity and more overall workout+ tweets. We also hypothesized that across both genders, the higher the Klout Score, the lower the minutes of shared physical activity.

**Figure 1:**
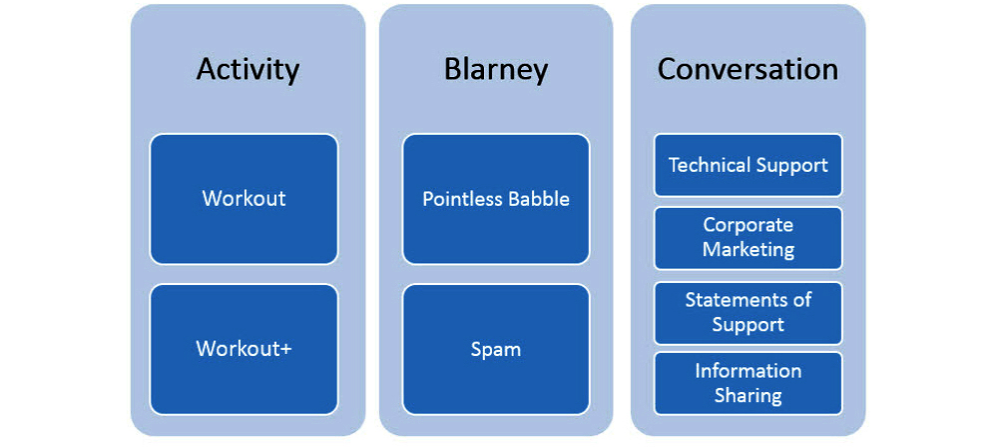
The fitness tweet classification model.

The data for this research were drawn from an existing dataset of fitness tweets from mobile fitness app users who shared their physical activity and, in some cases, additional conversation over Twitter. Over 184 days, 2,856,534 tweets were collected in 23 different languages. However, for the purposes of this study, only the English-language tweets were analyzed from the activity tweets, resulting in a total of 583,252 tweets.

The Fitness Tweet Classification Model [20] was used to classify each tweet into main categories of activity, blarney, and conversation and then into subcategories as shown in Figure 1.

The different types of collected information from the mobile fitness apps and each corresponding Twitter account provided a number of different and unique data points to review. For this experiment, those data points included the activity tweets, the user’s gender, the minutes of physical activity, and the user’s Klout Score. The statistical analysis of physical activity on Twitter from the four selected mobile fitness apps was performed in SAS 9.3, a software suite developed by the SAS Institute for advanced analytics, business intelligence, and predictive analytics, using two key datasets: (1) the first dataset included all user information about Twitter users who sent tweets relating to workout and workout+ and (2) the second dataset contained all the actual tweets sent by each user.

### Analysis 2: Sentiment Analysis

#### Recruitment

Of the activity tweets, there were a total of 408,574 workout+ tweets. From this total, a random sample of 23,391 was created. These tweets were user-generated, where the end user provided additional text to a workout tweet (ie, the user provided supplementary information beyond that which was created by the app itself). Tweets were then grouped by mobile fitness app using the corresponding hashtags. There were no significant numbers of emojis available in the fitness tweets for use in the sentiment analysis.

#### Sentiment Analysis of Tweets

The AYLIEN Text Analysis for Google Sheet add-on was utilized for the analysis of the sentiment for each collected information-sharing conversation tweet as filtered by the Fitness Tweet Classification Model.

The AYLIEN Tweet Sentiment Analysis function is a three-step process:

1. Preprocessing: tweets are normalized and reformatted, and the parts that are considered irrelevant to the sentiment are stripped.
2. Parsing: tweets are parsed and their structure, tags, and negations are extracted.
3. Classification: tweets are classified as positive, negative, or neutral by a pretrained classifier, assisted by a lexicon-based approach as a second judge.

For this experiment, the sentiment analysis tool that analyzed each tweet and returned the value of positive, neutral, or negative was used for classification. These data were saved into an Excel spreadsheet for additional data processing by converting the text value to a numerical value (positive=1, neutral=0, and negative=–1).

## Results

### Analysis 1: Measuring Online Influence

#### Gender Assignment of Twitter Users Within the Dataset

Twitter does not collect the gender of users. To be able to compare across genders, a means of identifying the possible gender of the Twitter users was needed. To accomplish this, we used the US Social Security Administration’s name database to match English names with gender. The name database from the Social Security Administration website included popular names ranked by gender since 1880.

The first gender-match calculation between the first names in the collected Twitter demographic database (the Twitter user’s full name was one of the many demographic characteristics collected from Twitter) and the Social Security Administration database eliminated names that were used fewer than 200 times because many such names were much more popular among one gender than another (eg, girls were named Aaron <0.5% of the time). The assumption was that this adjustment eliminated a vast majority of gender confusion among names. Once this was completed, names were matched to genders using the VLOOKUP function in Excel.

A second gender-match calculation was performed for those Twitter users with names that appeared less than 200 times, in which we attempted to assign gender to the remaining names that did not match in the first round. Usernames that did not match either gender (<2%) were not included in the analysis.

After gender assignment, a descriptive statistical analysis was performed to compute the frequency of the following: (1) total minutes by gender, (2) total minutes by Klout Score, (3) total minutes by gender and Klout Score, (4) total number of tweets, (5) minutes exercised per tweet, and (6) total number of workout and workout+ tweets (separately).

#### Determination of Klout Quartiles

To examine the distribution of tweets, minutes of exercise described by said tweets and the categories mentioned in each tweet (workout or workout+), it was necessary to separate the users’ Klout Scores into quartiles. We used the quartile method of data classification to create categories with a rank-ordered dataset split into four equal parts.

This was done through a two-step process in SAS. First, the distribution of Klout Scores was examined using the univariate procedure in SAS (PROC UNIVARIATE) and assigned quartiles based on that distribution. Second, using a data step, values of 1, 2, 3, and 4 were assigned to observations within the first, second, third, and fourth quartiles, respectively (Table 1). The maximum of any Klout Score is 100 and the minimum is 1. It was determined that the median Klout Score from the collected dataset was 20.50. As reported by Klout, the mean Klout Score is 40, with users with a score of 63 ranked in the 95th percentile [27].

**Table 1:** Klout Score quartiles.

Quartile	Klout Score
100% Maximum	100.00
99%	56.59
95%	49.03
90%	44.09
75% Q3	35.65
50% Median	20.50
25% Q1	11.92
10%	10.10
5%	10.00
1%	10.00
0% Minimum	1.00

#### Number of Activity Tweets (Male Versus Female)

The descriptive statistical analysis found that males produced 57.9% (336,109/583,252) of the total of activity tweets, whereas females produced 42.1% (247,143/583,252). This difference was consistent across Klout quartiles (Table 2).

#### Number of Tweets (Male/Female Among Workout Groups)

The descriptive analysis was expanded to compare males and females in the activity category. It was found that both genders tweeted far more among the workout group than the workout+ group (72.01%, 420,010/583,252 vs 27.99%, 163,242/583,252) in the lowest Klout quartile. This trend decreased slightly through the second and third Klout quartiles and then dramatically among the highest quartile of Klout Scores. In that quartile, the number of tweets varied much less (56.79%, 70,229/123,656 vs 43.21%, 53,427/123,656).

#### Mean Minutes Per Tweet (Males Versus Females)

The ANOVA procedure (PROC ANOVA) within SAS was used to compare the mean number of minutes tweeted by each gender using gender in the class statement and setting the model as minutes=gender. It was found that, overall, the mean number of minutes tweeted did not vary significantly between males and females. However, the mean number of minutes tweeted was almost double among females of the lowest Klout Score quartile (Klout ≤11.92).

#### Determination of Activity Tweets by Klout Quartile

After assigning quartiles, we examined the frequency of observations within each stratum of Klout Scores using PROC FREQ in SAS for the following (Table 3): (1) minutes by Klout Score quartile and (2) exercise types by Klout Score quartile.

#### Tests of Significance Between Groups: Minutes Tweeted Between Workout Categories

Also using the ANOVA procedure within SAS, analysis compared the total number of minutes tweeted among workout groups (workout vs workout+) and found a statistically significant difference (*P*=.01; Table 4).

### Analysis 2: Sentiment Analysis

#### Sentiment Analysis of Workout+ Tweets

In total, there were 23,391 unique tweets within the original dataset that fit the filtering criteria from this random sample. Four of the mobile fitness apps were used in this analysis: DailyMile, Endomondo, Nike+, and RunKeeper. The overall sentiment of all mobile fitness apps suggests that half of these workout+ activity tweets were neutral in nature (Table 5). In addition, there were four times as many positive tweets than negative. The breakdown of sentiment analysis for negative, neutral, and positive sentiment by mobile fitness apps is also presented in Table 5.

**Table 2:** Klout Score by activity tweet (N=583,252) and gender.

Quartile and Klout Score	Activity tweets, n (%)	
	Male (n=336,109)	Female (n=247,143)
1: ≤11.92 (n=179,831)	102,007 (56.7)	77,824 (43.3)
2: >11.93 and ≤20.50 (n=154,669)	89,822 (58.1)	64,847 (41.9)
3: >20.51 and ≤35.65 (n=125,096)	73,394 (58.7)	51,702 (41.3)
4: >35.65 (n=123,656)	70,886 (57.3)	52,770 (42.7)

**Table 3:** Workout and workout+ tweets by Klout quartile.

Quartile and Klout Score	Workout tweets (n=420,010)			Workout+ tweets (n=163,242)		
	Tweets, n	Minutes (total)	Minutes per tweet, mean (SD)	Tweets, n	Minutes (total)	Minutes per tweet, mean (SD)
1: ≤11.92	143,552	6,320,924	44.05 (97.26)	36,279	1,745,722	48.12 (128.83)
2: >11.93 and ≤20.50	118,047	5,125,345	43.42 (65.54)	36,622	1,666,997	45.53 (91.67)
3: >20.51 and ≤35.65	88,182	4,348,112	49.32 (324.43)	36,914	1,694,811	45.91, (104.47)
4: >35.65	70,229	2,897,436	41.26 (54.97)	53,427	2,550,963	47.75 (285.42)

**Table 4:** Minutes exercised by gender and Klout Score among workout group.

Quartile and Klout Score		Male			Female		
		Tweets (% total males)	Minutes (total)	Minutes per tweet, mean (SD)	Tweets (% total females)	Minutes (total)	Minutes per tweet, mean (SD)
**Workout^a^**		241,254	10,935,339	45.33 (48.10)	178,756	7,756,479	43.40 (96.69)
	1: ≤11.92	81,503 (33.78)	3,528,992	43.33 (48.10)	62,049 (34.71)	2,791,932	45.00 (137.26)
	2: >11.93 and ≤20.50	67,666 (28.05)	2,942,049	43.48 (56.45)	50,381 (28.18)	2,183,296	43.34 (76.06)
	3: >20.51 and ≤35.65	51,863 (21.50)	2,811,512	54.21 (420.74)	36,319 (20.32)	1,536,600	42.33 (51.54)
	4: >35.65	40,222 (16.67)	1,652,786	41.09 (49.08)	30,007 (16.79)	1,224,650	41.50 (61.62)
**Workout+^b^**		94,855	4,437,573	46.79 (234.49)	68,387	3,220,919	47.10 (117.44)
	1: ≤11.92	20,504 (21.62)	952,567	46.46 (114.94)	15,775 (23.07)	793,154	50.28 (144.89)
	2: >11.93 and ≤20.50	22,156 (23.36)	1,002,024	45.24 (85.01)	14,466 (21.15)	664,973	45.97 (101.02)
	3: >20.51 and ≤35.65	21,531 (22.70)	983,395	45.67 (112.10)	15,383 (22.49)	711,416	46.25 (98.06)
	4: >35.65	30,664 (32.33)	1,499,587	48.90 (362.80)	22,763 (33.29)	1,051,375	46.19 (117.88)

^a^ There was no significant difference between males and females in the number of tweets for workouts (*P*=.64).

^b^ There was no significant difference between males and females in the number of tweets for workout+ (*P*=.55).

**Table 5:** Total number of tweets by sentiment and app.

Tweets and sentiment	Total	DailyMile	Endomondo	Nike+	RunKeeper
Total number of tweets, n	23,391	9298	820	3999	9284
Positive sentiment, n (%)	9389 (40.14)	7097 (76.41)	211 (25.73)	418 (10.45)	1663 (17.91)
Negative sentiment, n (%)	2342 (10.01)	1392 (14.99)	51 (6.22)	350 (8.75)	549 (5.91)
Neutral sentiment, n (%)	11,660 (49.85)	799 (8.60)	558 (68.05)	3231 (80.80)	7072 (76.17)

**Figure 2:**
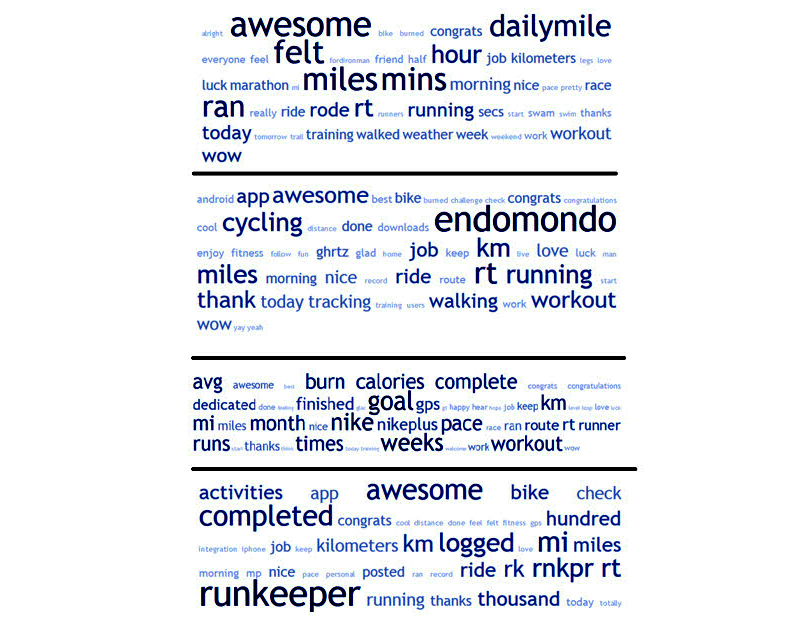
Word clouds by mobile fitness app.

## Discussion

### Analysis 1: Measuring Online Influence

This study further explored a novel approach to classify fitness tweets through Klout influence score. The study further stratified by gender through the use of a validated government database, which was probability matched to our data using exact matching procedures. This gender validation allowed for additional analysis of the gender breakdown of the existing dataset. The data were filtered through the matching criteria twice to improve precision, resulting in a 97% gender match. Although we gender matched twice, the process used to gender match could still be missing a few names that appear more often today than they did even a few years ago. Because popular names can change with high frequency, some gender matching in this study may not be valid within several years.

Based on the current database of collected fitness tweets from five mobile fitness apps, the highest Klout quartile included those individuals with a Klout Score of 35.65 or greater. Klout Scores can reach 100; therefore, our highest score tier may not capture an accurate representation of the most influential people on the Twitter platform. Additional insights from this research are described subsequently.

#### Men Share Their Physical Activity From Mobile Fitness Apps Via Twitter More Often Than Women

Based on this research, men share their workouts using Twitter and mobile fitness apps more often than women (54.35%, 336,109/618/458 vs 45.65%, 282,349/618,458). Although we believe this to be the first gender analysis of the sharing of physical activity from mobile fitness apps using Twitter, previous research on the overall gender use of Twitter suggests that more women than men use Twitter [28], with some nonacademic research suggesting that 40 million more women use Twitter on a monthly basis, that 62% of Twitter users are women [29], and those with a higher Klout Score tend to be women. Additional research regarding gender suggests that women are likely to be more active on Twitter as opposed to men, with women tweeting once every 20 hours versus men tweeting once every 26 hours [30].

However, additional research into our dataset using third-party software called Demographics Pro suggests that the average mobile fitness app user in the fitness tweet dataset is a male in his early thirties, typically married with children and having a high income. Additional insights into the users of mobile fitness apps who also tweet their physical activity includes that this group’s most common professions are programmers, photographers, church leaders, designers, and teachers. The group has a notably high concentration of Web developers (within the top 10% of overall Twitter distribution in this respect). In their spare time, they particularly enjoy beer, political news, wine, comedy/humor, and cooking. People in this group are charitably generous and particularly health conscious. Sports that rise most notably above the Twitter norm include cycling, skiing, and golf. As a consumer, this group is relatively affluent, with spending focused most strongly on technology, wining/dining, and health/fitness. Their strongest brand affiliations include Apple Store, Trader Joe’s, CrossFit, Trek Bicycle, and MyFitnessPal.

#### The Design of the Mobile Fitness App and the Sharing of Physical Activity Data to Social Networking Sites Matters

The sharing of workout+ tweets is dramatically enhanced by the user interface of the mobile fitness app. When comparing the four mobile fitness apps for the total number of activity tweets (workout tweets plus workout+ tweets), the most popular mobile fitness app was Endomondo (211,240 tweets), followed by NikePlus (203,991 tweets), DailyMile (183,732 tweets), and MyFitnessPal (70,723 tweets). The same usage ranking order was seen with men and women (men: 123,482 for Endomondo, 116,388 for NikePlus, 106,846 for DailyMile, and 70,723 for MyFitnessPal; women: 87,758 for Endomondo, 87,603 for NikePlus, 76,886 for DailyMile, and 30,233 for MyFitnessPal). However, there was a large difference when reviewing the workout+ tweets with 97.67% (173,790/177,943) of all workout+ tweets from DailyMile, 1.89% (3358/177,943) from NikePlus, 0.44% (776/177,943) from Endomondo, and no workout+ tweets from MyFitnessPal. In reviewing the user interface for all four mobile fitness apps, it is evident that the design of DailyMile made it much easier to share not only the workout, but also additional information about the workout when compared to the other three mobile fitness apps. Also during the evaluation time period for the activity tweets, Endomondo used a third-party service called @addthis to share workout+ tweets. With no workout+ tweets from MyFitnessPal, we determined that the app made a design decision to not allow users to share additional information regarding their physical activity workouts.

#### There is Brand Loyalty Regarding Mobile Fitness App Usage and the Sharing of Physical Activity Data Using Twitter

Of the 113,340 overall users in the dataset, 97.21% (110,186 users) tweeted their physical activity from just one mobile fitness app, 3105 (2.74%) used two mobile fitness apps, with 101 (0.09%) users sharing from three mobile fitness apps and just one user (0.0009%) sharing from four mobile fitness apps. We base this on the analysis of tweets per users and cannot determine the actual usage of the app, only the sharing of physical activity data from the apps. We surmise one reason that more than 97% used just one app could be loyalty, but other reasons such as poor user interface and difficulty in connecting one’s Twitter account to the mobile fitness app may account for other reasons.

### Analysis 2: Sentiment Analysis

A better understanding of the online influence of those who are sharing their fitness tweets may lead to new and innovative ways to encourage their followers to be more physically active through peer-to-peer influence, similar to programs created by marketing agencies to influence consumer behavior. Analogous to the other health-related research, physical activity researchers can monitor and attempt to influence physical activity Twitter chatter sent by influential Twitter users who are physically active and popular among various demographic groups and age ranges [15]. The findings can be used to inform online and offline efforts that work to target individuals who are most at risk for the harms associated with a lack of physical activity.

The relatively high number of neutral tweets was expected because each of the mobile fitness apps had a predetermined structure that limited additional information that could be included by the user. There also is the fact that a majority of the tweets simply did not contain words or phrases that could be classified as either positive or negative. Additional insights from this research are described subsequently.

#### The Real-Time Shared Sentiment of the Physical Activity Can Provide Additional Insights to Physical Activity

We believe that the sharing of one’s physical activity with additional commentary (for the purposes of this research called workout+ tweets) from mobile fitness apps can provide researchers with new insights that in the past may have been difficult to measure. The design of many of the mobile fitness apps allows for the user to share characteristics such as who they were with, the type of weather, the location of the physical activity, and their immediate thoughts regarding the physical activity. These and other insights will allow physical activity researchers to have a greater understanding into the real-time reasons, thoughts, and sentiment of how and perhaps why a person partakes in physical activity. These data will enable a greater understanding surrounding the complexities of physical activity, which can then be used for an enhanced design of mobile fitness apps as a potential tool in the decrease of physical inactivity.

#### Most Shared Mobile Fitness App Physical Activity Is of a Structured Exercise Type

It is through the analysis and interpretation that the context of fitness tweeting from within mobile fitness apps provides insights into what is being shared, by whom, and for what reasons. Based on the type of information collected, it can be expected that a majority of the activities shared using mobile fitness apps through Twitter were of a more structured exercise type, as opposed to continuous monitoring of daily physical activity. This is possibly due to the additional battery drain on the mobile phone of the user, which would preclude daylong usage of the app. In addition, the structure of the tweets would also suggest that these activities were measured in terms of duration, suggesting activities such as a run, walk, bike, or traditional workout. Because of the nature of some of the activity tweets, it was possible to extract additional information, including the actual type, distance, and the amount of time spent on an activity. It was possible for outliers to be present within the database. For example, the first use of a mobile fitness app could be the user testing the mobile fitness app that may have prompted an activity tweet with a very short-duration activity (seconds rather than minutes), whereas very long-duration activities were sometimes recorded for activities when the person did not properly end his or her mobile fitness app activity session. It was possible that some of the longer-duration activities were, in fact, long exercise sessions. For example, a person training for a marathon would track long runs.

#### A Significant Majority of Users From Each App Used the App More Than Once

Based on the research data, the number of one-time users of a mobile fitness app that shared their workout using Twitter (activity tweets) was calculated. Although the research cannot determine if a person continued to use a mobile fitness app and decided not to share via Twitter, it was determined that of all users, between 17% and 27% used the sharing to Twitter feature only once depending on the app. A number of reasons could exist for one-time use, including user error, experimentation of sharing functionality, or testing by a user choosing a mobile fitness app. From the 165,768 users that posted activity using a mobile fitness app that was then shared via Twitter, the database included 76,192,059 minutes of activity over the 6-month time period. These minutes are equivalent to 52,911 days, 1738 months, or more than 145 years of combined activity minutes. We cannot determine if this physical activity was the only performed physical activity by each user during the time period because it is understood that users may have completed physical activity without using their mobile fitness app.

These findings and interpretations should be regarded as exploratory and speculative because they represent what can be potentially done in a short development time and with ease of use for non-computer programming health-promotion researchers.

### Limitations

There are a number of limitations to this research study. Utilizing outside data, in this case the US government, to determine each user’s gender leaves room for error.

This research was conducted using the Twitter firehose, which allows for the collection of all publicly available tweets. Although we are confident in this data-collection process, there is no way to verify it without a financial expense to purchase all tweets. There also remains a challenge in the extraction of useful data from these repositories through data mining and knowledge discovery [6] due to a rapidly evolving explosion of data services and tools that can be used for analysis. This is due in large part to commercial pressures and the potential for using social networking data for computational research [31]. To minimize this limitation, we were able to link different datasets using the user’s Twitter name as the unique identifier through free publicly available data. Future work could enhance our model by purchasing commercially available datasets for analysis.

There has been a steady growth of social media usage, from 5% of the US population in 2005 to close to 70% in 2015. As more Americans have adopted social media, the user base has also grown more representative of the broader population; however, it is still most used by younger age groups [32].

### Comparison With Prior Work

The use of social media and emerging technologies to study physical activity and the possible lack thereof continues to increase with the development of such technologies. Previous research has shown an interest in specific characteristics of the social environments adversely affecting health outcomes [3]. Other research has studied the use of wearables and other smart devices to quantify various different health conditions with the self-reported data being shared on social networks, such as Facebook and Twitter [9], and have suggested that the adoption of such emerging technology to monitor physical activity has created new research opportunities to observe, quantify, and define physical activity in the real-world setting [2]. Our research continues to build on these previous studies by providing researchers with other options for data collection and different objectives to consider.

Previous work regarding the role of technology on physical activity through social media includes a dearth of studies that have studied various aspects of the impact of social media on physical activity. Some research has focused on the behavior change challenges that include self-monitoring, goal setting, and problem-solving strategies [33]. Other research has suggested a change in how we think about physical activity and sedentary behavior measurement, a research topic that includes the use of mobile fitness apps and social networks that can collect large amounts of real-time data that previously would have been difficult to collect [34]. Research by Tsoh [35] explores contextual and psychological factors that may underlie the observed low physical activity levels among mobile fitness app users. Our research is more closely related to that of Grundy et al [36] on the network analysis of prominent health and fitness apps and work by Haddadi et al [37] on the integration of shared health and fitness data from mobile fitness apps that are shared over social networks. Although these works are highly relevant to the research presented in this paper, we expand the research by carrying out data analysis including gender and online influence.

Similar approaches to inferring gender include works using a gender-based dictionary [38], through profile picture and background inference [39], and a third-party Web service that can often reveal gender through proprietary algorithms [40]. Specific research on using social media networks and physical activity include work by Althoff et al [41] on the influence of Pokemon Go, the tweeting of physical activity as a possible method to increase physical activity by Tsoh [35], and work by Liu and Young [42] on using social media data analysis for physical activity surveillance.

### Future Work

We created a very powerful tool for conducting large-scale research by collecting physical activity data from Twitter, but the demographics used in this research could suggest a bias regarding the breakdown of mobile fitness app users and thus underrepresent certain groups. If researchers wish to use Twitter and mobile fitness apps for physical activity research, additional steps would need to be taken to ensure that all groups are represented in the data samples collected. Apart from technical limitations, there could be ethical challenges that are equally as challenging. Although tweets are considered public, they may contain information that many would consider “private” due to the possible misconception of the perceived audience (a user’s Twitter followers) versus the actual audience (data researchers) [9]. To expand on this work, additional investigation could address possible trends specific to forms of physical activity per gender that could constitute a higher Klout Score.

The popularity of consumer-facing health wearables (eg, Fitbit, Garmin) that also share physical activity data with online social networks would be a topic worthy of future research. By using these tracking devices, which monitor physical activity on an ongoing basis, a more inclusive picture of daylong physical activity can be achieved. This is in contrast to mobile fitness app data, which is typically collected and shared following a traditional “workout” (eg, a walk, run, bike). The same data collection and classification model presented in this paper can be used with minimal changes. With regards to online influence, other work could use an alternate measure of online influence rather than Klout.

### Conclusion

This research analyzed publicly shared physical activity data collected via Twitter from five different mobile fitness apps. From this dataset, two analyses on the data were conducted to highlight the unique ability to use this type of data within the study of physical activity. The first analysis categorized the users into four quartiles that represented their online influence as calculated by Klout as well as a method to assign gender to each Twitter user. The analysis suggests that men share their workout tweets more than women, that there is more basic sharing of physical activity data (workout tweets) when compared to tweets that also contain commentary by the user (wourkout+ tweets), and that there is no significant difference in the tweeting of men and women. The second analysis was conducted with workout+ tweets and showed, across all apps, most of the shared tweets were neutral, but for those with a sentiment there were four times as many positive tweets as negative.

## References

[1] Lewis BA, Napolitano MA, Buman MP, Williams DM, Nigg CR (2017). Future directions in physical activity intervention research: expanding our focus to sedentary behaviors, technology, and dissemination. J Behav Med.

[2] Schrack JA, Cooper R, Koster A, Shiroma EJ, Murabito JM, Rejeski WJ, Ferrucci L, Harris TB (2016). Assessing daily physical activity in older adults: unraveling the complexity of monitors, measures, and methods. J Gerontol A Biol Sci Med Sci.

[3] Schootman M, Nelson EJ, Werner K, Shacham E, Elliott M, Ratnapradipa K, Lian M, McVay A (2016). Emerging technologies to measure neighborhood conditions in public health: implications for interventions and next steps. Int J Health Geogr.

[4] Garimella V, Weber I (2017). A long-term analysis of polarization on Twitter. http://arxiv.org/abs/1703.02769.

[5] (2017). Twitter.

[6] Arias M, Arratia A, Xuriguera R (2013). Forecasting with Twitter data. ACM Trans Intell Syst Technol.

[7] Pagoto S, Schneider KL, Evans M, Waring ME, Appelhans B, Busch AM, Whited MC, Thind H, Ziedonis M (2014). Tweeting it off: characteristics of adults who tweet about a weight loss attempt. J Am Med Inform Assoc.

[8] Driscoll K, Walker S (2014). Big data, big questions - working within a black box: transparency in the collection and production of big Twitter data. Int J Commun.

[9] Wang Y, Weber I, Mitra P (2016). Quantified self meets social media: sharing of weight updates on Twitter.

[10] Khan FH, Bashir S, Qamar U (2014). TOM: Twitter opinion mining framework using hybrid classification scheme. Decis Support Syst.

[11] Bravo-Marquez F, Mendoza M, Poblete B (2014). Meta-level sentiment models for big social data analysis. Knowledge-Based Syst.

[12] Liu B, Indurkhya Nitin (2010). Sentiment analysis and subjectivity. Handbook of Natural Language Processing.

[13] Broniatowski D, Paul M, Dredze M (2013). National and local influenza surveillance through Twitter: an analysis of the 2012-2013 influenza epidemic. PLoS One.

[14] Tsuya A, Sugawara Y, Tanaka A, Narimatsu H (2014). Do cancer patients tweet? Examining the twitter use of cancer patients in Japan. J Med Internet Res.

[15] Cavazos-Rehg PA, Krauss M, Fisher SL, Salyer P, Grucza RA, Bierut LJ (2015). Twitter chatter about marijuana. J Adolesc Health.

[16] Freifeld CC, Brownstein JS, Menone CM, Bao W, Filice R, Kass-Hout T, Dasgupta N (2014). Digital drug safety surveillance: monitoring pharmaceutical products in twitter. Drug Saf.

[17] Bollen J, Mao H, Zeng X (2011). Twitter mood predicts the stock market. J Comput Sci.

[18] Pagoto SL, Schneider KL, Oleski J, Smith B, Bauman M (2014). The adoption and spread of a core-strengthening exercise through an online social network. J Phys Act Health.

[19] Vickey T, Breslin J, Tsai N (2011). Mobile fitness apps and Twitter-a systemic review. Proceedings of the 8th International Symposium on Computer Science in Sport.

[20] Vickey TA, Ginis KM, Dabrowski M (2013). Twitter classification model: the ABC of two million fitness tweets. Transl Behav Med.

[21] Hansen D, Shneiderman B, Smith M (2009). Analyzing Social Media Networks: Learning by Doing with NodeXL.

[22] Lassen D, Brown A (2010). Twitter. Soc Sci Comput Rev.

[23] Quercia D, Ellis J, Capra L, Crowcroft J (2011). In the mood being influential on Twitter mood.

[24] Miller K (2011). Wall Street Journal.

[25] Barbieri S, Feltracco P, Omizzolo L, Snenghi R, El Mazloum R, Vettore G, Bergamini M, Stefanati A, Donato D, Ferronato C, Avato FM, Tredese A, Gaudio RM (2017). Planking or the “lying-down game”: two case reports. Interact J Med Res.

[26] Edwards C, Spence PR, Gentile CJ, Edwards A, Edwards A (2013). How much Klout do you have…a test of system generated cues on source credibility. Comput Human Behav.

[27] (2013). The Klout Score.

[28] Duggan M, Greenwood S, Perrin A (2016). Pew Research Center.

[29] McCandless D (2012). Huffington Post.

[30] Hargittai E, Litt E (2011). The tweet smell of celebrity success: explaining variation in Twitter adoption among a diverse group of young adults. New Media Soc.

[31] Batrinca B, Treleaven PC (2014). Social media analytics: a survey of techniques, tools and platforms. AI Soc.

[32] (2017). Pew Research Center.

[33] King AC, Hekler EB, Grieco LA, Winter SJ, Sheats JL, Buman MP, Banerjee B, Robinson TN, Cirimele J (2016). Effects of three motivationally targeted mobile device applications on initial physical activity and sedentary behavior change in midlife and older adults: a randomized trial. PLoS One.

[34] Kelly P, Fitzsimons C, Baker G (2016). Should we reframe how we think about physical activity and sedentary behaviour measurement? Validity and reliability reconsidered. Int J Behav Nutr Phys Act.

[35] Tsoh JY (2016). Tweeting about physical activity: can tweeting the walk help keeping the walk?. Mhealth.

[36] Grundy Q, Held FP, Bero LA (2017). Tracing the potential flow of consumer data: a network analysis of prominent health and fitness apps. J Med Internet Res.

[37] Haddadi H, Ofli F, Mejova Y, Weber I, Srivastava J (2015). 360-Degree quantified self.

[38] Liu W, Ruths D (2013). What's in a name? Using first names as features for gender inference in Twitter.

[39] Alowibdi J, Buy UA (2013). Language independent gender classification on Twitter.

[40] An J, Weber I (2016). #greysanatomy vs #yankees: Demographics and hashtag use on Twitter. https://arxiv.org/pdf/1603.01973.pdf.

[41] Althoff T, White RW, Horvitz E (2016). Influence of Pokmon Go on physical activity: study and implications. J Med Internet Res.

[42] Liu S, Young S (2016). A survey of social media data analysis for physical activity surveillance. J Forensic Leg Med.

